# Functionalization of magnetic nanoparticles by creatine as a novel and efficient catalyst for the green synthesis of 2-amino-4H-chromene derivatives

**DOI:** 10.1038/s41598-022-14844-0

**Published:** 2022-06-23

**Authors:** Reza Eivazzadeh-Keihan, Shahrzad Bahrami, Mostafa Ghafori Gorab, Zahra Sadat, Ali Maleki

**Affiliations:** Catalysts and Organic Synthesis Research Laboratory, Department of Chemistry, Iran University of Science and Technology, Tehran, 16846-13114 Iran

**Keywords:** Materials science, Chemistry, Catalysis, Heterogeneous catalysis

## Abstract

By employing the naturally-originated molecule of creatine, Fe_3_O_4_@SiO_2_-creatine as an environmentally benign magnetic organometallic nanobiocatalyst was successfully prepared via a convenient and green route. Then to acquire an inclusive comprehension of different properties of the catalyst, it was studied by various characterization techniques such as FT‐IR, FE-SEM, TEM, EDX, XRD, and VSM analyses. It was found that the size distribution of nanoparticles was an average diameter size of 70 nm. To examine the catalytic activity, it was applied in sequential knoevenagel condensation-Michael addition room temperature reaction of dimedone, malononitrile, and different substituted aromatic aldehydes to produce a variety of 2-amino-tetrahydro-4*H*-chromene-3-carbonitrile derivatives in a single step. Among the multiple outstanding advantages that can be mentioned for this work, some of the most noticeable ones include: affording the products in short reaction times with high yields, operating the reaction at ambient conditions and ease of catalyst separation.

## Introduction

In the last decades, due to growing concerns about environmental contamination and its extensive detrimental effect on earth's ecosystems, designing more environmentally benign chemical processes has attracted substantial attention. Global interest has recently shifted into developing more eco-friendly synthetic approaches to accomplish the most important goal of green chemistry which is stopping or decreasing the use and generation of hazardous and toxic chemicals^1–3^. In this regard, great efforts have been made to the use of bio-based feed stocks such as cellulose^4,5^, chitosan^6,7^, alginic acid^8,9^ and newly, creatine as natural supports in designing and producing different catalysts^10^. Creatine is a naturally occurring nitrogen-containing organic acid that exists primarily in muscle tissue of vertebrates and plays a very significant role in the energy supply process for muscle tissue by participating in recycling adenosine triphosphate (ATP) via giving a phosphate group to adenosine diphosphate (ADP)^10^. Creatine’s natural production in the human body mainly takes place in the liver and kidneys and includes the enzyme-promoted reaction between glycine and arginine amino acids to form guanidinoacetate^11^. Then, in the second step, enzyme-assisted methylation of guanidinoacetate using s-adenosyl methionine as the methyl-group donor produce creatine. After that, the as-synthesized creatine is being transferred to the muscles through the bloodstream. Apart from the major bio-synthesized supply of creatine, it can also be obtained from the diet^12^. Since creatine is naturally originated, it doesn’t have any harmful effect on living systems and affords the demand of chemists to employ green and natural materials. Having both carboxylic and amine groups, creatine is capable of activating raw materials both in acid-catalyzed reactions as well as base-catalyzed reactions; so, it has attracted chemist’s attention as a potential bifunctional catalyst. Herein, by supporting this biocompatible, non-toxic and biodegradable molecule on a magnetic core, a novel reusable organometallic superparamagnetic creatine-based nanobiocatalyst was designed, prepared, characterized and then it was employed in a one-pot three-component condensation reaction for the synthesis of 2-amino-tetrahydro-4*H*-chromene-3-carbonitrile. In addition to have a broad spectrum of biological and pharmacological properties such as antitumor^13,14^, antibacterial^15–17^, antiviral^18^, antifungal^19^, anti-influenza^20^, anti-inflammatory^21^, anticancer^22,23^, and antiallergenic activity^24,25^, these oxygen-containing heterocyclic scaffolds exhibits a lot of industrial applications in the field of cosmetics and pigments^26^, laser dyes^27^, photoactive materials^28^, optical brighteners, fluorescent markers^29^ and biodegradable agrochemicals^30,31^. Due to their wide range of applications, there exist several reports of employing homogeneous and heterogeneous catalysts to promote the synthesis of 2-amino-tetrahydro-4*H*-chromene-3-carbonitrile frameworks^32–42^. Although all of these catalysts offer some advantages, many of them suffer from different defects such as the requirement of energy inputs like microwaves or ultrasonic irradiation or high temperature, using of toxic and expensive solvents, catalysts and reagents, long reaction times, high catalyst loading, tedious workup procedures, and low yields. Our green efficient protocol for production of 2-amino-tetrahydro-4*H*-chromene-3-carbonitriles consist of three-component catalytic reaction between malononitrile, an aldehyde, and dimedone and is catalyzed by above-mentioned nanobiocatalyst. Through bio-functionalizing the surface of magnetic nanoparticles (MNPs) with creatine, the core–shell Fe_3_O_4_@SiO_2_-creatine nanocatalyst was successfully prepared. Recently a large number of researches have been focused on magnetic nanoparticles because of their remarkable features like providing high surface, facilitating catalyst separation and product purification and lot of other merits^43,44^. Due to the magnetic characteristic of this creatine-functionalized catalyst, it can be easily collected from the reaction mixture by a magnet bar which eliminates the possibility of the presence of remained catalyst particles in the pharmaceutical final products.

## Experimental

### General

All solvents, chemicals, and reagents were purchased from Merck, Sigma and Aldrich. Thin-layer chromatography (TLC) was used to monitor the progress of catalytic reactions. Melting points were measured with an Electrothermal 9100 apparatus. FT‐IR spectra were recorded on a Shimadzu IR‐470 spectrometer using KBr pellets. Elemental analysis of the nanocatalyst was carried out by EDX analysis recorded on Numerix JEOL-JDX 8030 (30 kV, 20 mA). XRD pattern of nanocomposite was recorded on an X′ Pert Pro X-ray diffractometer operating at 40 mA current and 40 kV. ^1^H and ^13^C NMR spectra were recorded on a Bruker DRX‐500 Avance spectrometer at 500 and 125 MHz, respectively. The morphology and structure of the nanocatalyst were examined by FE-SEM, MIRA3 TESCAN. TEM measurements were carried out on a Zeiss-EM10C-100 kV analyzer to prove the core–shell structure of the nanocomposite. The magnetic properties of the samples were detected at room temperature using a VSM of Meghnatis Daghigh Kavir.

### Synthesis of the nanobiocatalyst

The preparation of the nanobiocatalyst was carried out using simple and readily available precursors via four steps including the synthesis of Fe_3_O_4_ core, coating the magnetic core by silica shells, immobilizing creatine as an external shell, respectively.

#### Synthesis of Fe_3_O_4_ NPs

Fe_3_O_4_ NPs were synthesized via co-precipitation method^45^. Typically, 4.70 g FeCl_3_·6H_2_O and 1.72 g FeCl_2_·4H_2_O (with a molar ratio of 2:1) were mixed with 80 mL of distilled H_2_O in a round bottom flask under N_2_ atmosphere. The temperature was gradually increased up to 80 °C and at this point, 10 mL NH_3_·H_2_O (25% v/v) was added dropwise to the vigorously stirring above-mentioned mixture. After the addition of ammonia, stirring of the mixture was continued for 45 min and then it was cooled to room temperature. The resulting black sediment was aggregate with an external magnet and was frequently washed with distilled water, ethanol and acetone in turn and dried at 70 °C in an oven in order to make it ready for further modifications.

#### Synthesis of Fe_3_O_4_@SiO_2_ NPs

Coating the magnetic Fe_3_O_4_ core with a layer of SiO_2_ was performed through a modified Stöber method^46^. Initially, 2.00 g of as-prepared Fe_3_O_4_ NPs were dispersed in a mixture of 160 mL ethanol and 40 mL distilled water for 15 min using an ultrasonic water bath. Later, 10 mL of ammonia solution (25 wt%) was injected dropwise to the reaction mixture under intensive magnetic stirring. In the next step, 1 mL of TEOS was dropped slowly into the solution by a syringe for 20 min. in the last step, the mixture allowed to stir for 12 h at room temperature. Finally, the brown obtained solid was collected by a magnet and after successive washing with distilled water and ethanol several times it was dried at 60 °C.

#### Preparation of chloropropyl-functionalized Fe_3_O_4_@SiO_2_ MNPs

Binding (3-chloropropyl)-trimethoxysilan (CPTMS) as a linker to silica-coated MNPs was performed following the procedure of one of previous works^47^. Firstly, 1 mL of CPTMS reagent was dissolved in 100 mL of dried toluene. afterward, 1.00 g of Fe_3_O_4_@SiO_2_ was added to this mixture and the solution stirring last for 18 h at 60 °C. The resulted brown precipitate (Fe_3_O_4_@SiO_2_‒Cl) was washed with toluene, separated by a permanent magnet, and vacuum dried at 70 °C.

#### Synthesis of Fe_3_O_4_@SiO_2_-creatine

At first, 1.00 g of as-prepared Fe_3_O_4_@SiO_2_–Cl nanoparticle and 1.31 g (10 mmol) of creatine were added to 80 mL of ethanol and the mixture was stirred in a round bottom flask under refluxing conditions for 10 h. Then, the obtained solid substance was magnetically separated, washed repetitively with distilled water, ethanol and acetone and dried at 70 °C in an oven.

#### General procedure for the synthesis of 4*H*-chromene derivatives (4a–o)

First, 1.00 mmol (0.14 g) dimedone, 1.00 mmol of aromatic aldehydes and 1.10 mmol (0.07 g) malononitrile were mixed together in 2 mL of ethanol in presence of 0.04 g of the prepared catalyst. This mixture stirred for appropriate times at room temperature. The progress of the reaction was monitored by TLC (ethyl acetate: n-hexane, 1:1). After completion of the reaction, the catalyst was separated using an external magnet and the obtained precipitates which were the products, recrystallized in ethanol to gain highly pure crystalline 4*H*-chromene compounds.

## Results and discussion

**Figure 1:**
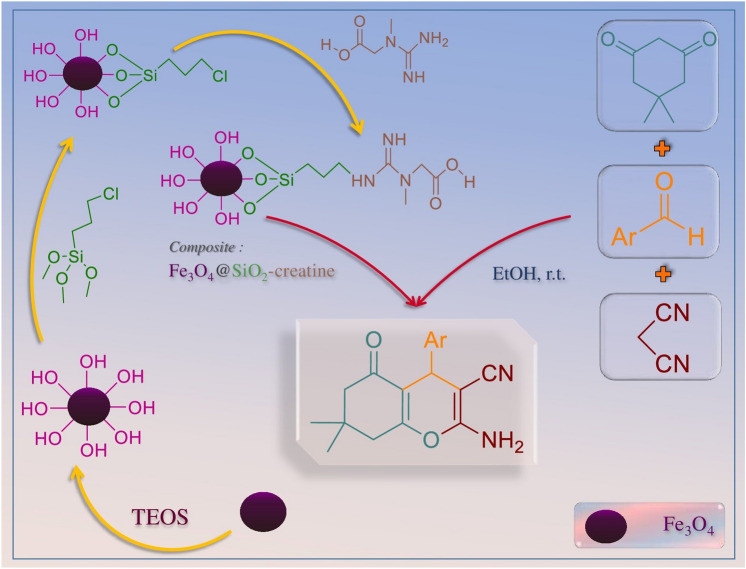
Preparation of creatine-functionalized Fe_3_O_4_@SiO_2_ NPs and synthesis of 4H-chromene derivatives (4a–o).

In this study, a unique bifunctional nanobiocatalyst with acidic and basic sites was designed by immobilizing creatine on the surface of Fe_3_O_4_@SiO_2_ NPs. At first, Fe_3_O_4_@SiO_2_ NPs were synthesized. In the second step, these NPs were modified by biomolecule of creatine as an environmentally friendly and low-cost compound (Fig. 1). Several identification techniques such as FT-IR, EDX, FE-SEM, TEM, and VSM were employed to study the properties of the nanocatalyst. Also, after several times using it as a bifunctional catalyst for the synthesizing of 2‐amino‐4*H*‐chromenes, there was no specific changing in the structure and activity of the mentioned catalyst.

### Characterization of the nanocatalyst

#### FT-IR spectroscopy

**Figure 2:**
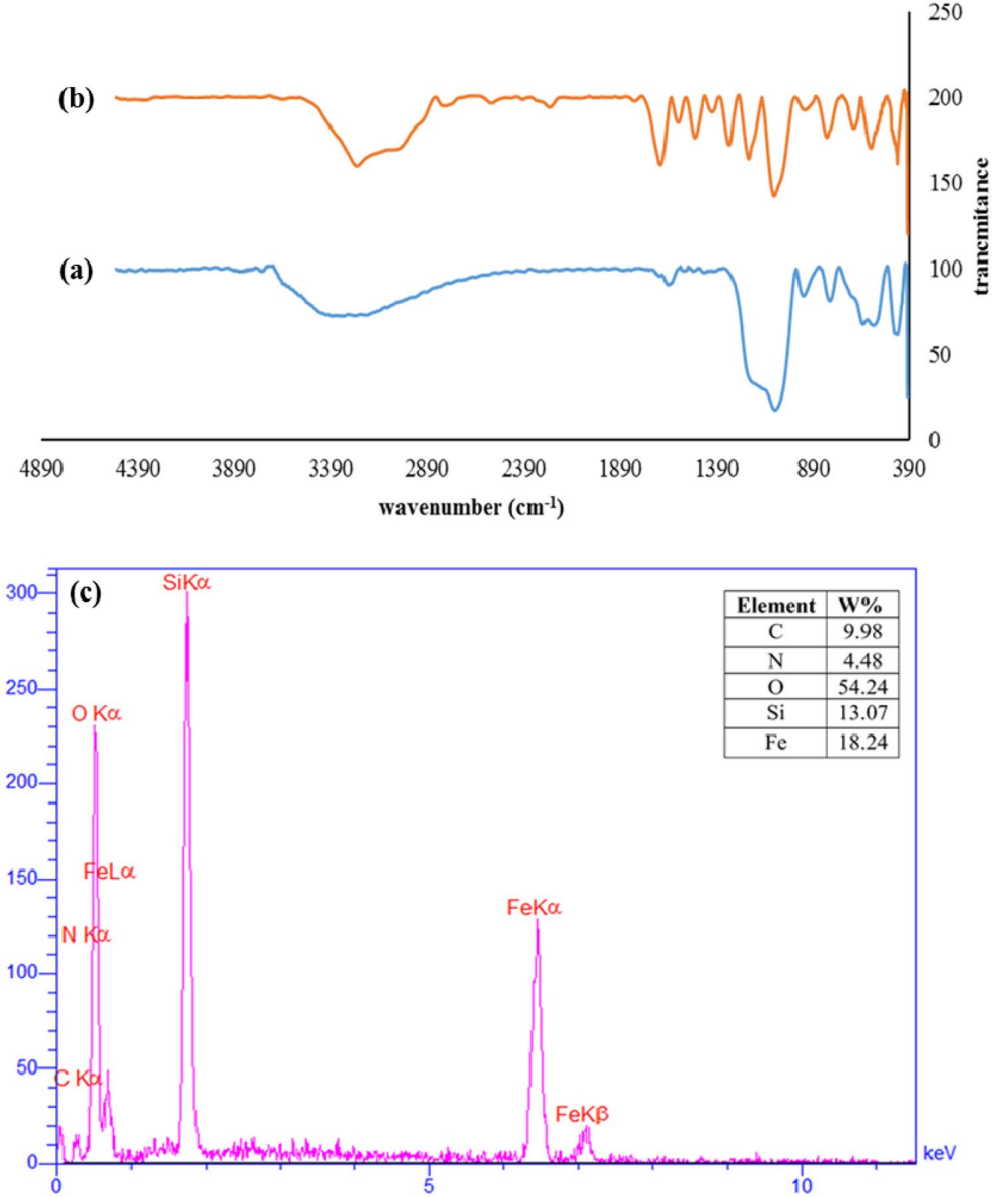
FT-IR spectra of (**a**) chloropropylated Fe_3_O_4_@SiO_2_ MNP, (**b**) Fe_3_O_4_@SiO_2_-creatine nanobiocomposite and (**c**) EDX analysis of Fe_3_O_4_@SiO_2_-creatine nanobiocomposite.

FT-IR spectra of Fe_3_O_4_@SiO_2_–Cl and the final creatine-coated MNPs are depicted in Fig. 2a,b. As can be seen in the spectrum Fig. 2a, the peak appeared at 576 cm^−1^ is ascribed to Fe^2+^–O–Fe^2+^ and the one at 632 cm^−1^ to Fe^3+^–O–Fe^3+^ stretching vibrations^48,49^. The sharp band appearing at 1089 cm^−1^ is attributed to asymmetric stretching vibration and the one at 802 cm^−1^ symmetric stretching vibrations of Si–O–Si bond^50^. The absorption peaks of the Fe_3_O_4_@SiO_2_–Cl at 2894 cm^−1^ and 2964 cm^−1^ are related to the symmetric and asymmetric stretching vibration of C–H bonds^44^. Additionally, peaks appeared at about 3300 cm^−1^ are assigned to the asymmetric stretching vibration of hydroxyl groups on the surface of the silica layer. Coating of magnetic core with the organo-layer of creatine is confirmed by stretching vibrations appeared at 1683 cm^−1^ which is related to C=O functional group of creatine biomolecule (Fig. 2b)^51^. Peaks appeared in the IR spectrum of the composite are very good in agreement with functional groups existing in the structure of the organo-functionalized MNPs and could be counted as evidence to approve the core–shell structure of as-described nanocatalyst.

#### EDX analysis

EDX analysis was performed to determine the elements in the composition of the catalyst. The result of EDX analysis is demonstrated in Fig. 2c. It clearly indicated the existence of iron, silicon, carbon, oxygen and nitrogen elements in the nanobiocomposite. The presence of the Fe peaks in the EDX result indicates the existence of Fe_3_O_4_ nanoparticles in the structure. In addition, silicon and nitrogen peaks are clear signs of TEOS and creatine, respectively. Carbon and oxygen peaks are related to the presence of these elements in TEOS and creatine molecules. The observed oxygen peak is also related to Fe_3_O_4_ nanoparticles.

#### FE-SEM image study

**Figure 3:**
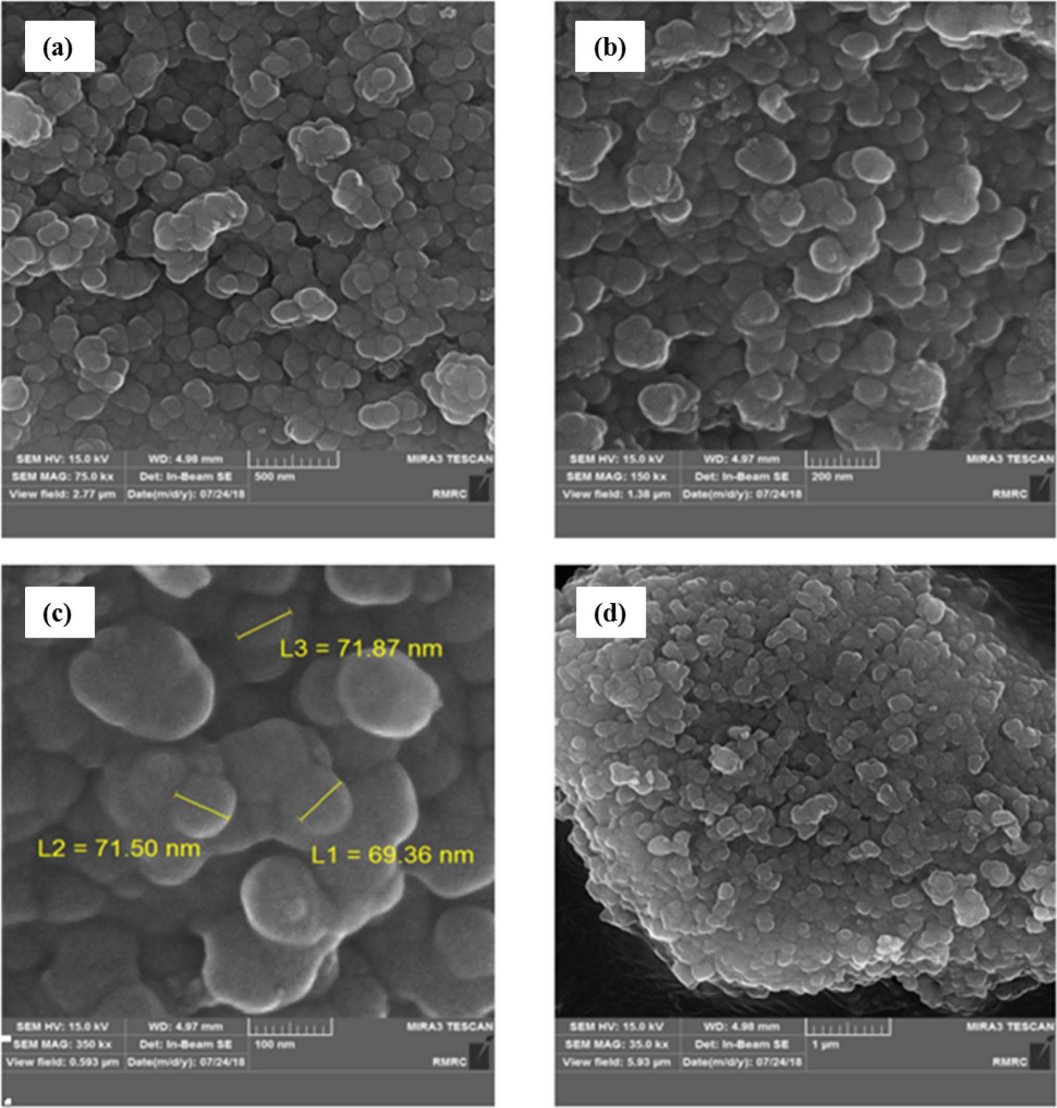
SEM images of Fe_3_O_4_@SiO_2_-creatine nanobiocatalyst in four different magnifications: (**a**) 500 nm, (**b**) 200 nm and (**c**) 100 nm and (**d**) 1 μm.

In order to identify the surface morphology and particle size distribution of the nanocatalyst FE-SEM imaging technique was used (Fig. 3a–d). As can be observed in SEM micrographs in four different magnifications, the Fe_3_O_4_@SiO_2_-creatine nanoparticles have an approximately spherical shape and size distribution with an average diameter length of 70 nm.

#### TEM image study

**Figure 4:**
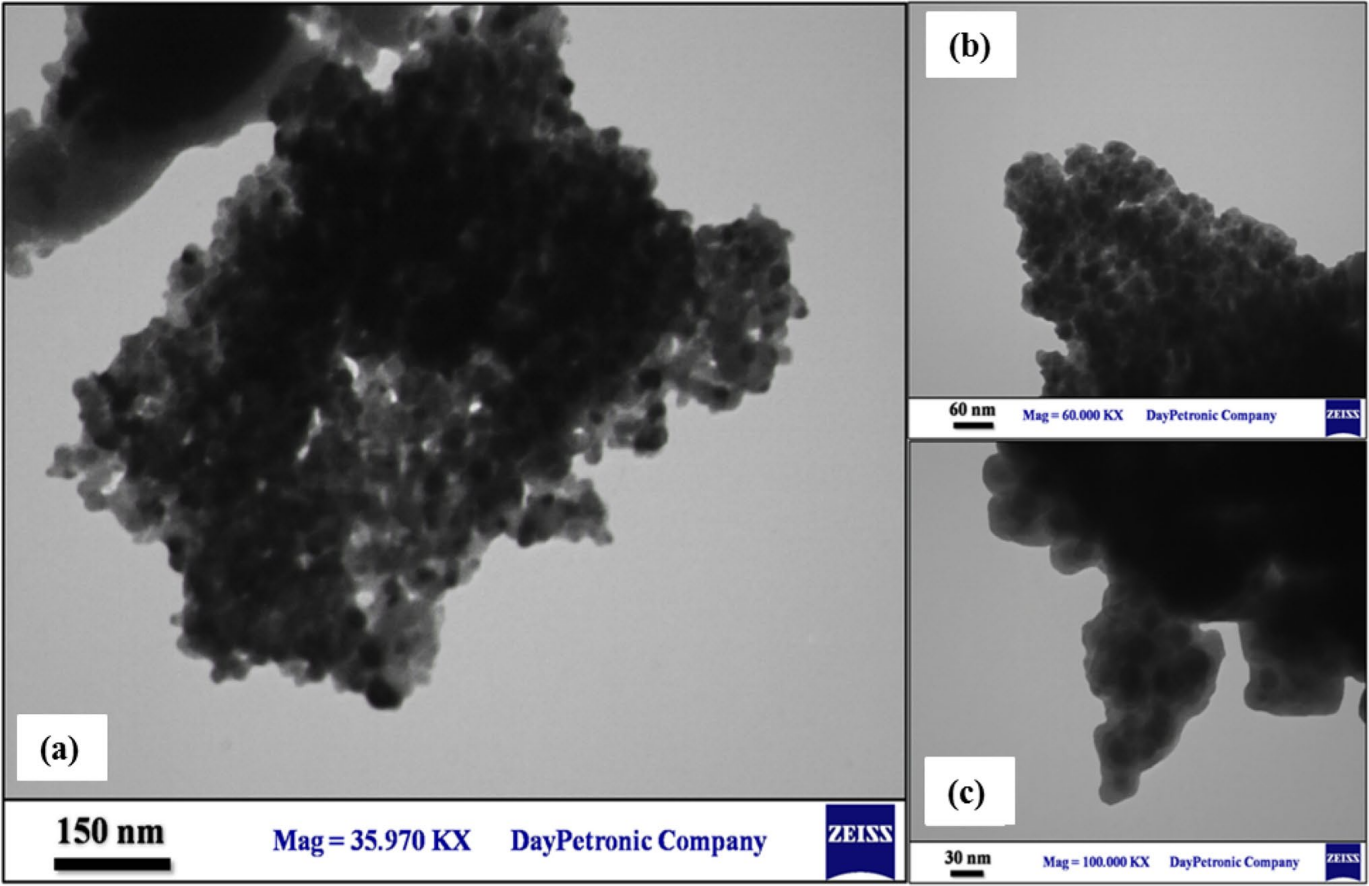
TEM images of Fe_3_O_4_@SiO_2_-creatine nanobiocatalyst in three different magnifications: (**a**) 150 nm, (**b**) 60 nm and (**c**) 30 nm.

To determine the structure of interior layers of the synthesized nanobiocatalyst and to prove the core–shell structure of it, TEM analysis was utilized. As it is observable in Fig. 4. TEM images were taken in 3 different magnifications 150 nm (Fig. 4a), 60 nm (Fig. 4b), 30 nm (Fig. 4c) which clearly show the shell consisting of silica inorganic layers and organic layer of creatine that have coated Fe_3_O_4_ core.

#### VSM analysis

**Figure 5:**
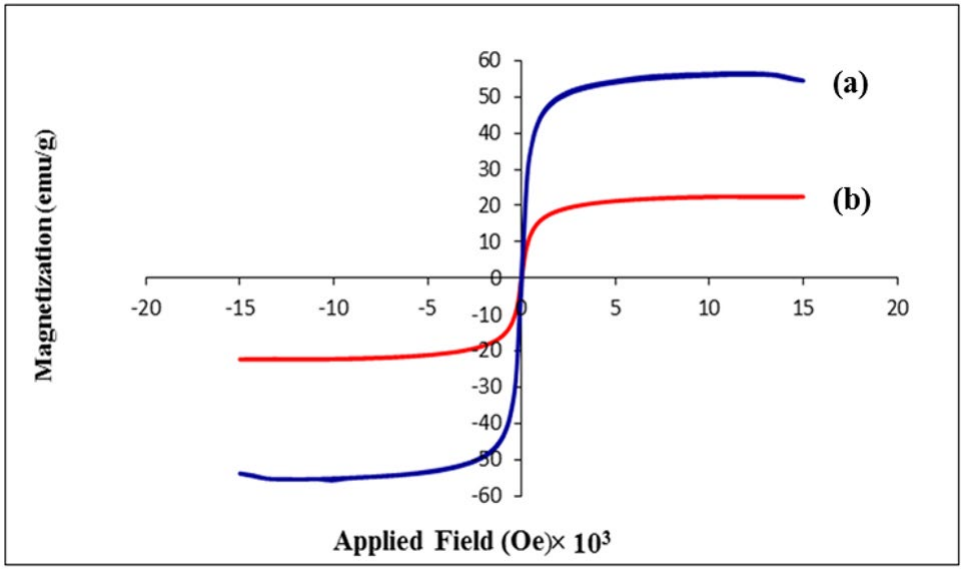
VSM magnetization curve of: (**a**) Fe_3_O_4_ and (**b**) Fe_3_O_4_@SiO_2_-creatine.

Magnetic characteristic of the Fe_3_O_4_@SiO_2_-creatine nanocatalyst was measured via VSM analysis at room temperature (300 K). Figure 5 shows the magnetization as a function of magnetic field strength. The saturation magnetization value (Ms) of uncoated MNPs (Fe_3_O_4_) (Fig. 5a) was found to be 56.0 emu g^−1^^52^. With sweeping the magnetic field from − 20,000 to + 20,000 oersted, the saturation magnetization value (Ms) of the prepared nanocomposite was found to be about 22.5 emu g^−1^ (Fig. 5b), which is lower than neat Fe_3_O_4_ NPs. This reduction in the saturation magnetization is mostly ascribed to the existence of shells that have wrapped the Fe_3_O_4_ core. Despite this reduction in the saturation magnetization of catalyst, the intensity of its magnetic power is still high and can be easily separated from the reaction media by an external magnet. Moreover, the resultant hysteresis loop clearly certifies the superparamagnetic characteristic of the prepared nanocomposite.

#### X-ray diffraction (XRD)

**Figure 6:**
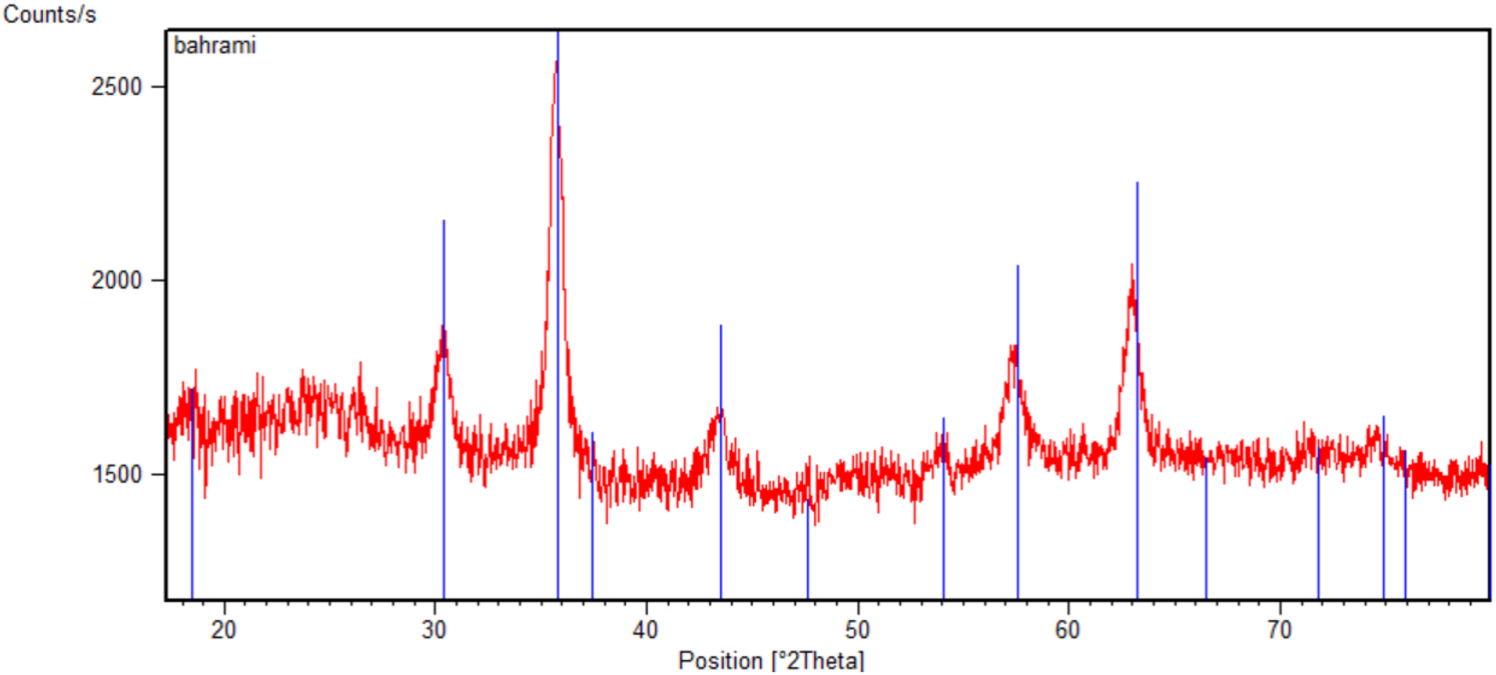
XRD pattern of Fe_3_O_4_@SiO_2_-creatine nanobiocatalyst.

The XRD pattern of the synthesized nanocatalyst in comparison with that of pure Fe_3_O_4_ is studied in a range of 5–80° and the resulting diffraction pattern is depicted in Fig. 6.

Characteristic diffraction peaks of the catalyst observed at 2θ = 18.38°, 30.32°, 35.70°, 43.33°, 53.82°, 57.38°, 62.95°, 74.31° respectively correspond to (1 1 1), (2 2 0), (3 1 1), (4 0 0), (4 2 2), (5 1 1), (4 4 0), (5 3 3) crystal planes of pure Fe_3_O_4_ with cubic inverse spinel structure. The location and relative intensities of all diffraction peaks are in good agreement with the standard XRD pattern of magnetite (JCPDS card No.01-075-0449), representing that cubic crystalline spinel structure of Fe_3_O_4_ have not changed during functionalization with creatine. Moreover, the broad peak from 2θ = 20° to 27° is referred to amorphous silica phase in the catalyst shell which can be declared as another proof that shows silica layers have successfully coated Fe_3_O_4_ NPs.

### Application of the nanobiocatalyst in organic synthesis

In order to investigate the catalytic activity of the created nanocatalyst, it was utilized in the one-pot synthesis of 2-amino-7,7-dimethyl-5-oxo-4-aryl-hexahydro-4*H*-chromene-3-carbonitrile derivatives. To optimize the reaction condition, the reaction between dimedone **3**, 4-isopropylbezaldehyde **2**, and malononitrile **1** (with a mole ratio of 1:1:1.1) in 2 mL of ethanol as a green solvent and at room temperature, was chosen as the model reaction.

At first, the effect of catalyst amount on the reaction rate and yield was studied. Using 0.04 g of the nanocatalyst was found to be adequate to catalyze the reaction and produce high yields of 4*H*-chromene derivatives. According to Table 1, in the presence of lower amounts of catalysts, lower yields of the products were obtained and the reaction took a longer time to complete. With increasing the amount of nanocatalyst no significant improvement in the reaction rate and yield was observed. As shown in Table 1, only trace amounts of the desired products were obtained in the absence of the nanocatalyst. To optimize the solvent of the reaction, the effect of H_2_O as another green solvent has also been studied. Finally, based on the results, ethanol was found to be the most suitable solvent (Table 1, entry 7). Since the desired products were produced in high to excellent yield at room temperature, testing higher temperatures were not needed.

**Table 1:** Optimizing the reaction conditions in the synthesis of 2‐amino‐4*H*‐chromene derivatives.

Entry	Catalyst loading (g)	Solvent	Temp (°C)	Time (min)	Yield^a^ (%)
1	–	–	r.t	60	Trace
2	–	–	80	60	25
3	–	EtOH	r.t	60	20
4	0.01	EtOH	r.t	45	45
5	0.02	EtOH	r.t	30	60
6	0.03	EtOH	r.t	20	80
7	0.04	EtOH	r.t	12	91
8	0.05	EtOH	r.t	12	91
9	0.04	H_2_O	r.t	15	85
10	0.04	–	r.t	30	70

^a^The yields refer to the isolated product **4h**.

In order to compare the efficiency of the prepared catalyst with its substrate before coating with creatine, the model reaction was performed with 0.04 g of each of the catalysts mentioned in Table 2. As can be seen in the table, at a specified time the efficiency of the prepared catalyst is much more than the substrates alone. Fe_3_O_4_ allows the catalyst to be easily separated from the reaction medium by an external magnet. In addition, Fe_3_O_4_ as Lewis acid can increase the reaction rate. Creatine as a proton donor agent activates electrophiles and also modifies nucleophilic reactions in the mechanism. The nitrogen in the structure of nanobiocatalyst acts as a base and improves the nucleophilic reaction of malononitrile and aldehyde by separating the acidic hydrogen from malononitrile. The synthesized nanobiocatalyst improves the reaction conditions by having a large number of acid–base parts.

**Table 2:** Comparison of catalytic activity of the prepared catalyst with the substrates alone.

Entry	Catalyst	Yield^a^ (%)
1	Fe_3_O_4_	40
2	Fe_3_O_4_@SiO_2_	45
3	Creatine	60
4	Fe_3_O_4_@SiO_2_-creatine	91

^a^Isolated yield.

In order to show the repeatability of this approach and to generalize the optimum conditions to other aromatic aldehyde a wide range of substituted benzaldehydes bearing both electron-withdrawing and electron-donating groups were chosen and the reactions of those aldehydes (**2**) with malononitrile (**1**) and dimedone (**3**) has led to the formation of a diverse sort of 2‐amino‐4*H*‐chromene-3-carbonitrile derivatives under optimum reaction conditions. The results presented in Table 3 show all products were successfully synthesized in good‐to‐excellent yields after suitable reaction time. Moreover, the results indicated that aldehydes possessing electron-withdrawing groups reacted much faster and gave higher yields than those bearing electron-releasing groups, however, the yield of the products with aldehyde bearing electron-releasing groups was completely satisfying in comparison with previous works^53–55^.

**Table 3:** Synthesis of 2‐amino‐4*H*‐chromene derivatives using creatine-based catalyst.


Entry	Aldehyde	Product	Time (min)	Yield^a^ (%)	Mp (°C)		
					Observed	Literature	
1	4-nitrobenzaldehyde	**4a**	5	96	176–182	176–183^56^	
2	3-nitrobenzaldehyde	**4b**	4	96	204–207	204–206^57^	
3	2-chlorobenzaldehyde	**4c**	4	96	212–215	213–215^58,59^	
4	3-chlorobenzaldehyde	**4d**	7	92	228–232	229–232^60^	
5	4-chlorobenzaldehyde	**4e**	6	94	214–217	214–216^59^	
6	3-methylbenzaldehyde	**4f**	10	88	197–200	198–200^61^	
7	4-methylbenzaldehyde	**4g**	10	86	215–219	215–218^62^	
8	4-isopropylbenzaldehyde	**4h**	12	91	196–200	198–200^61^	
9	2-methoxybenzaldehyde	**4i**	12	84	202–204	203–207^56^	
10	3-methoxybenzaldehyde	**4j**	8	86	197–200	197–199^63^	
11	4-methoxybenzaldehyde	**4k**	10	86	194–197	194–196^64–66^	
12	3,4,5-trimethoxybenzaldehyde	**4l**	9	91	175–177	175–179^67^	
13	3-hydroxybenzaldehyde	**4m**	15	82	233–235	232–234^68^	
14	4-hydroxybenzaldehyde	**4n**	18	81	208–210	208–210^69^	
15	2,4-dichlorobenzaldehyde	**4o**	6	94	118–120	118–120^70^	

^a^Isolated yields.

**Figure 7:**
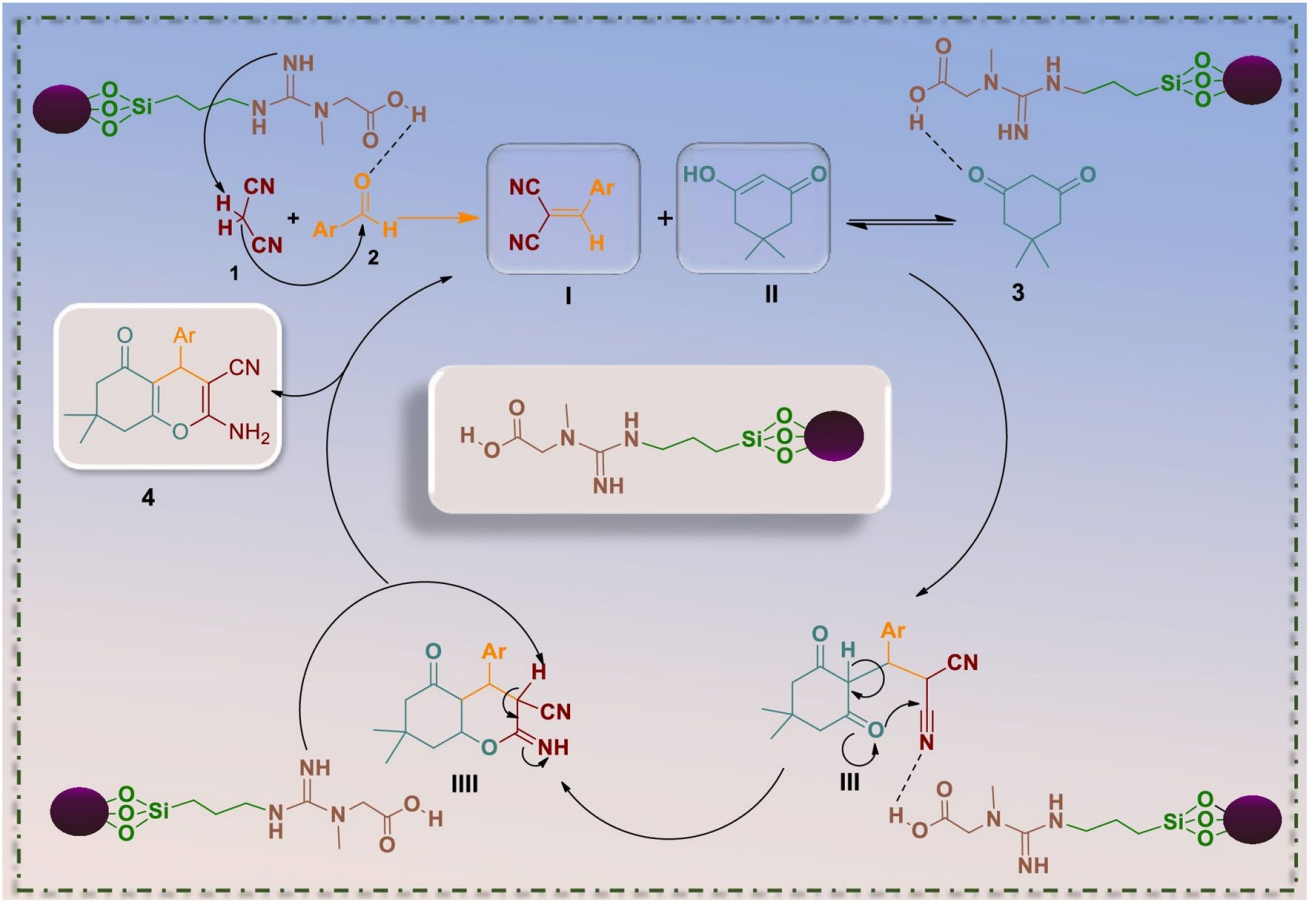
Proposed mechanism for the synthesis of 2‐amino‐4*H*‐chromene derivatives in presence of Fe_3_O_4_@SiO_2_-creatine.

The suggested possible mechanism of the reaction in the presence of the nanocatalyst is described in Fig. 7. Creatine as a bifunctional molecule can significantly catalyze the chromene formation reaction. Initially, the acidic hydrogen atom of the catalyst activates the aromatic aldehyde through protonation of its carbonyl group. So, the electrophilicity of the carbonyl group increases. Concurrently, the Nitrogen atoms in the guanidine part of the creatine (basic site of the catalyst) activates malononitrile via removing one of its acidic hydrogen atoms^71^. Then, the occurrence of a Knoevenagel condensation through Nu attack of active malononitrile carbanion to activated aldehyde along with excretion of a water molecule forms arylidene malononitrile intermediate (I). In the next step, tautomerization of dimedone takes place by the acidic part of the catalyst, and intermediate (II) is prepared. Then Michael addition of the enolized form of dimedone (II) to Knoevenagel adduct (I) is accomplished and intermediate (III) is prepared. Subsequently, the acidic part of the catalyst forms a hydrogen bond with nitrogen of CN, and by increasing the electrophilic property of CN, cyclization is done. At this step, intermediate (IV) is formed. Eventually, the basic site of the catalyst separates an acidic hydrogen from intermediate (IV) and the desired product is formed.

In addition, the catalytic activity of Fe_3_O_4_ @ SiO_2_-creatine was evaluated in comparison with other catalysts, and the results are shown in Table 4. As can be seen, the Fe_3_O_4_@SiO_2_-creatine catalyst performs better than other studies.

**Table 4:** Comparison of the catalytic ability of Fe_3_O_4_@SiO_2_-creatine with previous reports for synthesis of **4c**.

Entry	Catalyst	Solvent	Reaction time	Yield (%)	References
1	Melamine	H_2_O/EtOH (3;2)	25 min	93	^72^
2	Aminopropylated sillica gel	H_2_O	90 min	89	^73^
3	–	2,2,2-trifluoroethanol	5 h	85	^74^
4	Nano SiO_2_	H_2_O	10 min	90	^75^
5	2-aminopyridine	EtOH	8 min	92	^76^
6	MNPs·GO-CysA	H_2_O/EtOH (3;1)	15 min	95	^77^
7	Fluoride ion	H_2_O	30 min	84	^78^
8	Fe_3_O_4_@SiO_2_-creatine	EtOH	4 min	96	This research

### Catalyst recyclability

**Figure 8:**
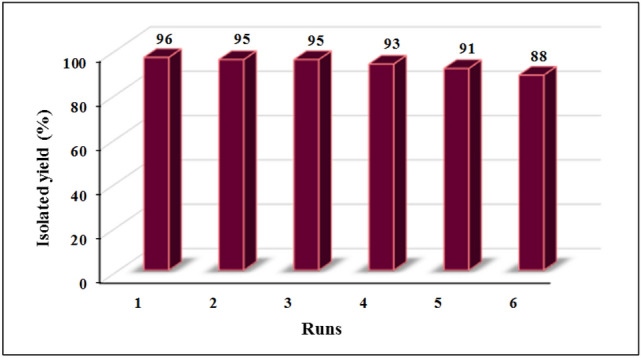
Reusability of Fe_3_O_4_@SiO_2_-creatine nanobiocatalyst for the synthesis of **4c**.

The reusability of the catalyst was assessed in the synthesis of 4*H*-chromene derivatives under optimum reaction conditions. Owing to its magnetic nature, the catalyst was easily separated from the reaction mixture by an external magnet, washed repeatedly with ethanol and distilled water, dried and reused after each run. It was observed that the catalyst could be reused for at least six times without any significant loss in its activity (Fig. 8). The result of EDX analysis (Fig. S19) and FT-IR spectrum (Fig. S20) of the reused catalyst showed no change in the composition of the catalyst.

## Conclusions

In summary, a novel and green renewable creatine-functionalized magnetic nanobiocomposite catalyst was designed and prepared via a facile, simple and cost-effective method beginning from readily accessible and non-toxic starting materials. Subsequently, in order to investigate the catalytic activity of the prepared nanocomposite, it was employed in the one-pot 3-CR synthesis of biologically-active 2-amino-7,7-dimethyl-5-oxo-4-aryl-hexahydro-4*H*-chromene-3-carbonitrile derivatives. The products were obtained with the yields of 81–96% within a few minutes. Multiple characterization techniques such as FT-IR, EDX, FE-SEM, XRD, TEM, and VSM were used to analyze various properties of the as-synthesized organocatalyst. The FE-SEM images showed that the average size of NPs was about 70 nm. The result of other applied identification techniques, all confirmed the formation of the core–shell spherical structure of the nanocatalyst. The advantages of this efficient environmentally-friendly protocol include ease of preparation and separation, providing safe and green reaction conditions, high atom economy and excellent yields in relatively short reaction times, magnetically recyclability of the heterogeneous catalyst, clean, simple and easy work-up procedure.

## Supplementary Information

Supplementary Information.

## Data Availability

The datasets used and/or analysed during the current study available from the corresponding author on reasonable request.
