# Supplementary material for: Functionalization of magnetic nanoparticles by creatine as a novel and efficient catalyst for the green synthesis of 2-amino-4H-chromene derivatives
Source: Sci Rep. 2022 Jun 23;12:10664. doi: 10.1038/s41598-022-14844-0 (PMC9226349; doi:10.1038/s41598-022-14844-0)
Supplement: Supplementary file 1 — Supplementary Information. [file 41598_2022_14844_MOESM1_ESM.docx]

**Supplementary Information**

**Functionalization of magnetic nanoparticles by creatine as a novel and efficient catalyst for the green synthesis of 2-amino-4H-chromene derivatives**

Reza Eivazzadeh-Keihan, Shahrzad Bahrami, Mostafa Ghafori Gorab, Zahra Sadat, Ali Maleki*

*Catalysts and Organic Synthesis Research Laboratory, Department of Chemistry, Iran University of Science and Technology, Tehran 16846-13114, Iran*

*^*^Corresponding author. E-mail:* [*maleki@iust.ac.ir*](mailto:maleki@iust.ac.ir)


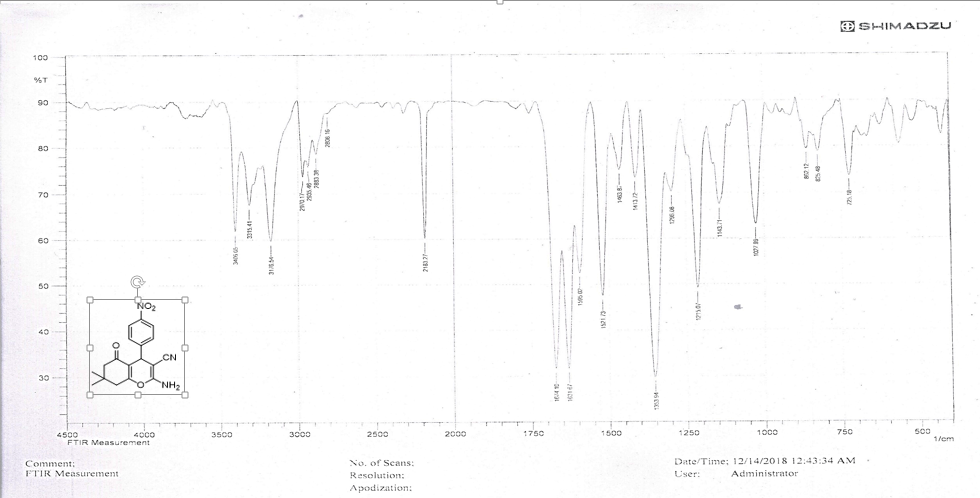


**Figure S1.** FT-IR spectrum of product (**4a**)


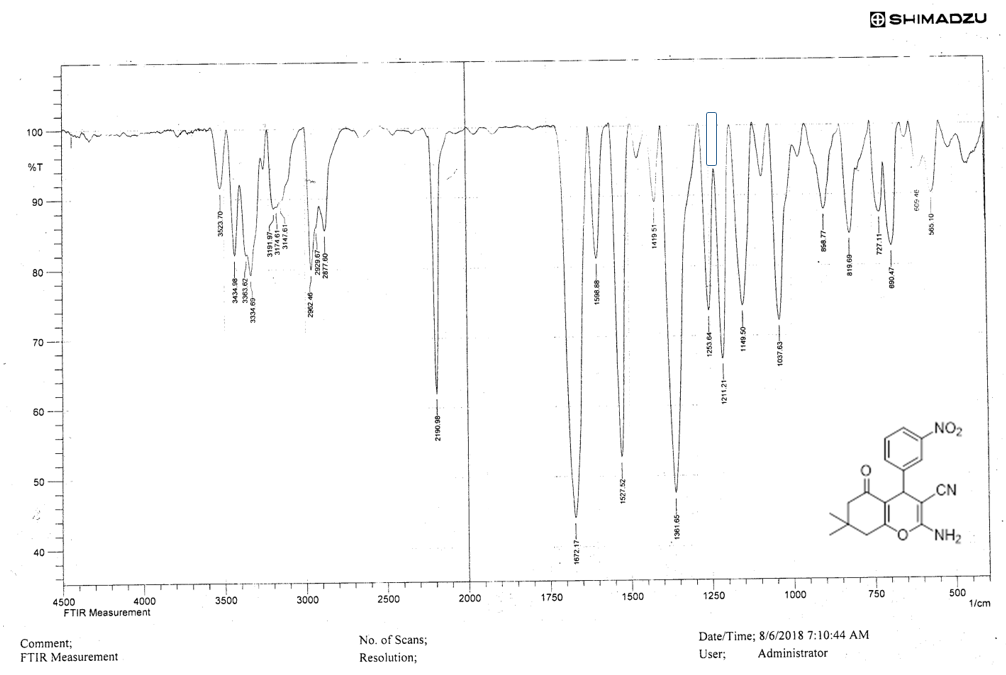


**Figure S2.** FT-IR spectrum of product (**4b**)


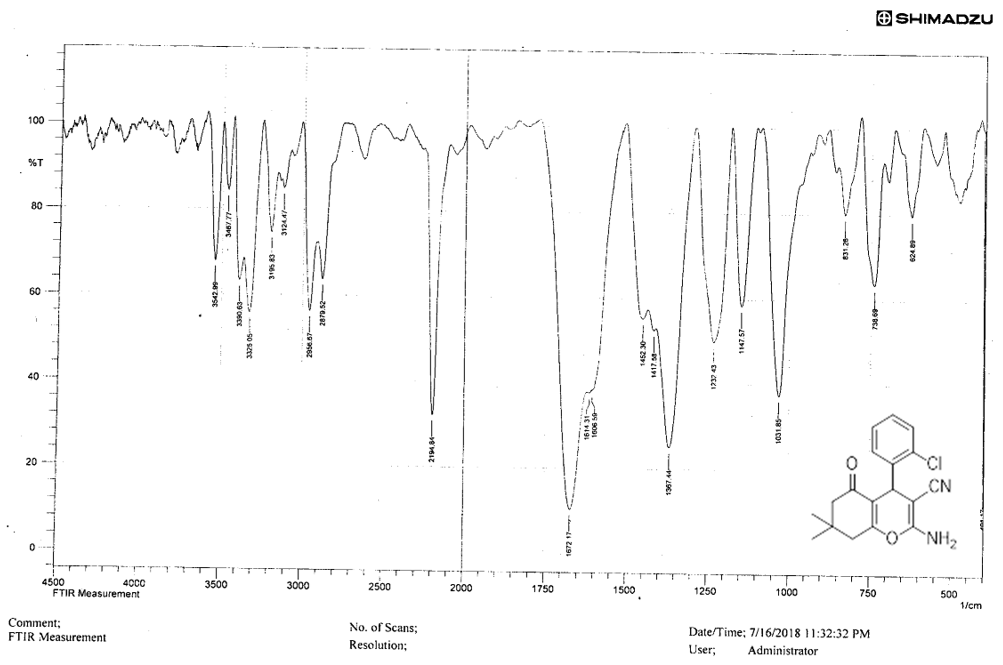


**Figure S3.** FT-IR spectrum of product (**4c**)


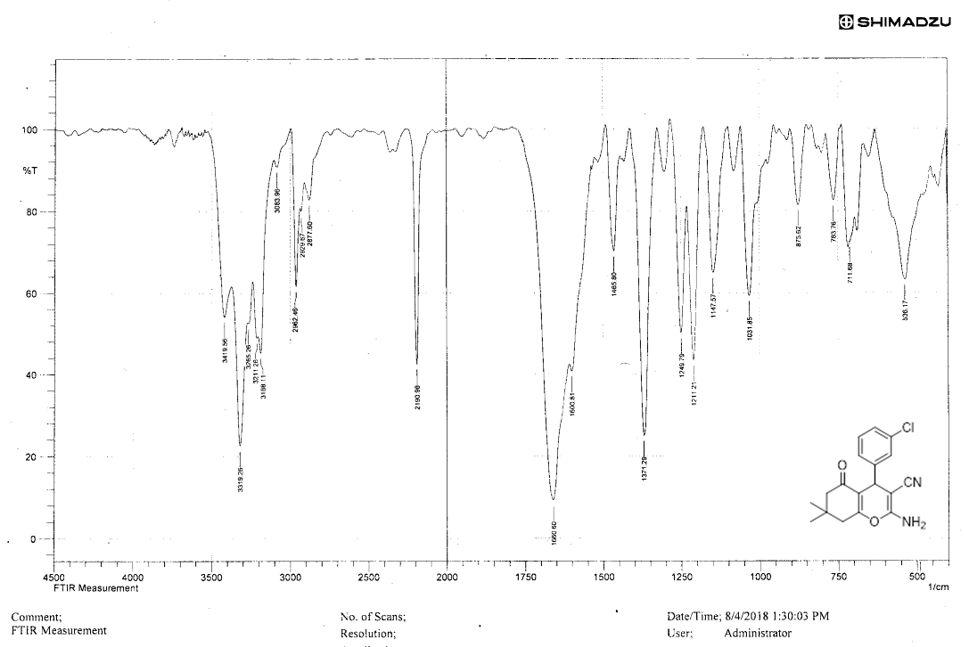


**Figure S4.** FT-IR spectrum of product (**4d**)


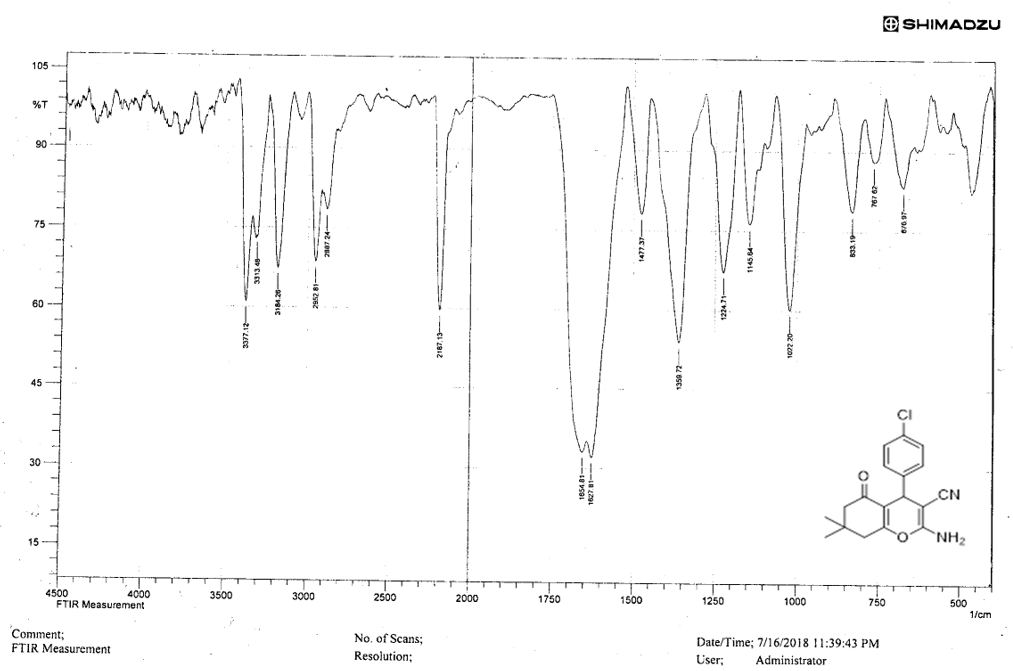


**Figure S5.** FT-IR spectrum of product (**4e**)


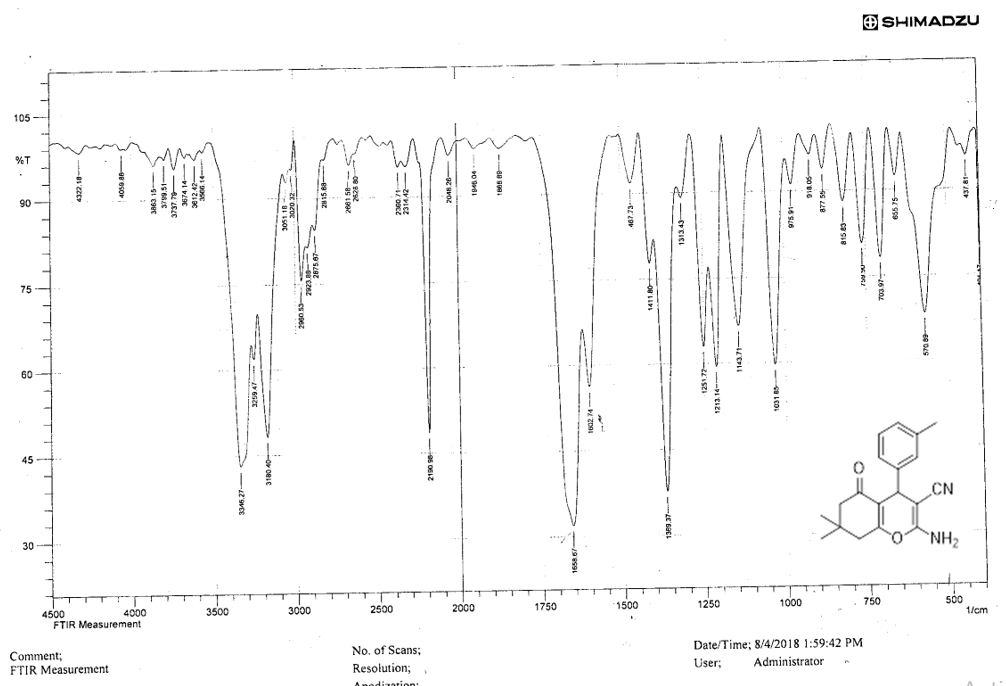


**Figure S6.** FT-IR spectrum of product (**4f**)


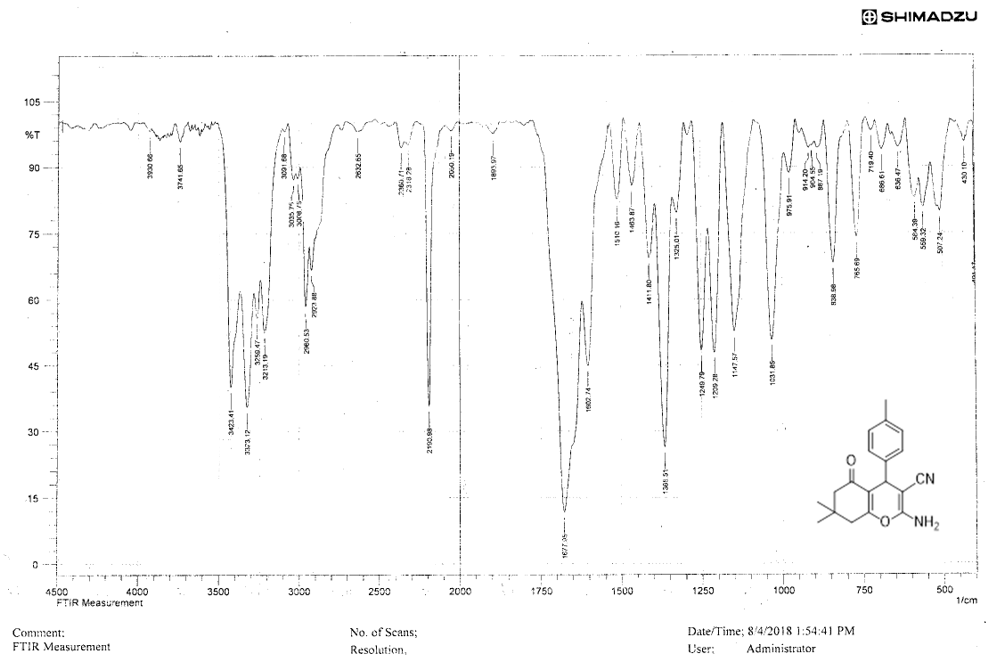


**Figure S7.** FT-IR spectrum of product (**4g**)


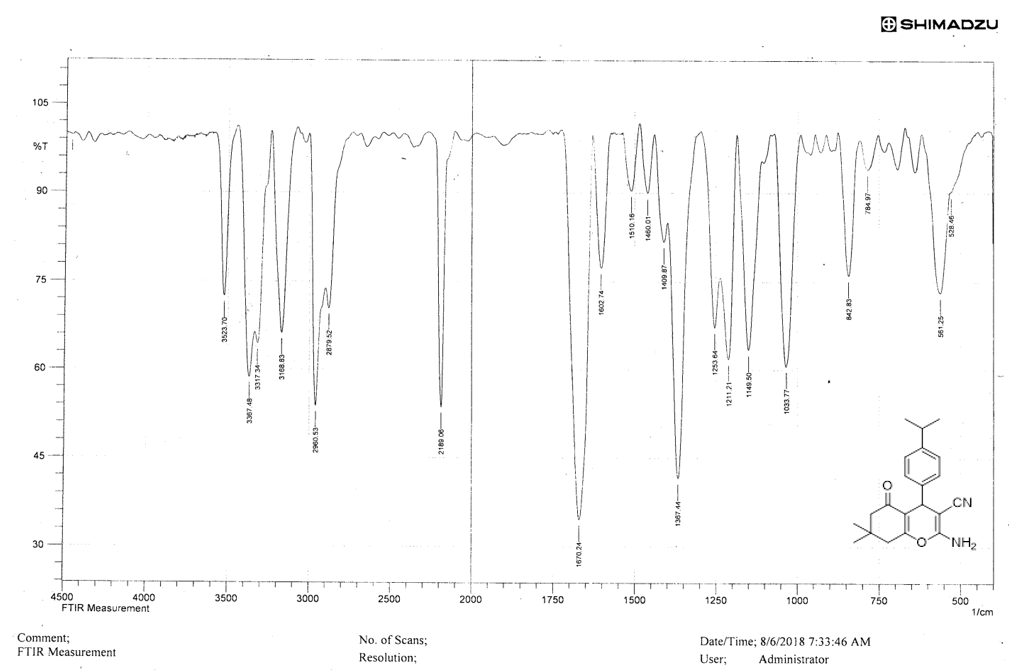


**Figure S8.** FT-IR spectrum of product (**4h**)


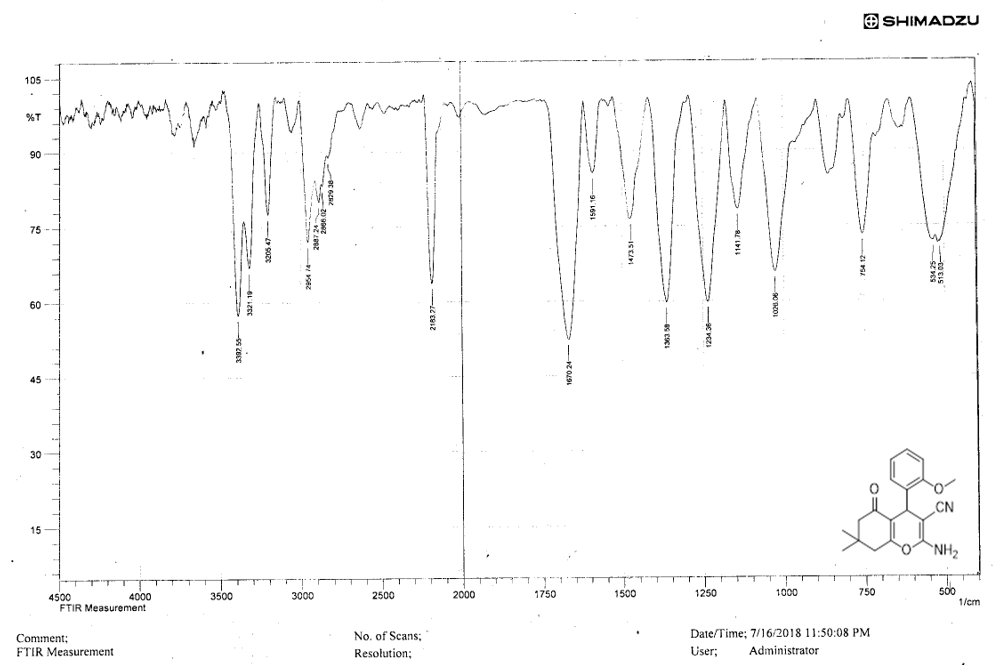


**Figure S9.** FT-IR spectrum of product (**4i**)


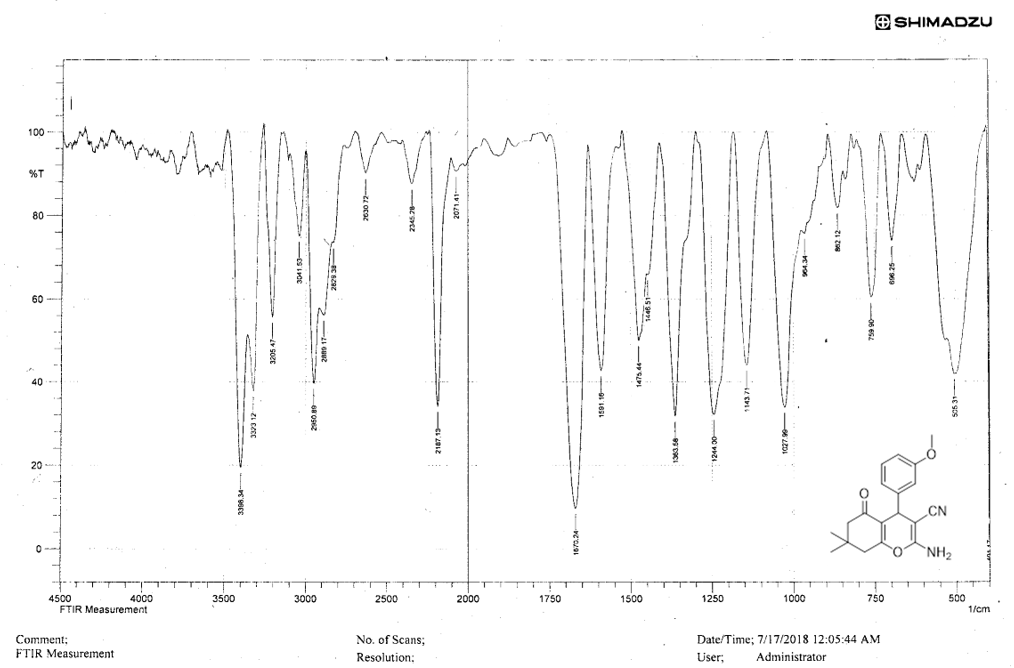


**Figure S10.** FT-IR spectrum of product (**4j**)


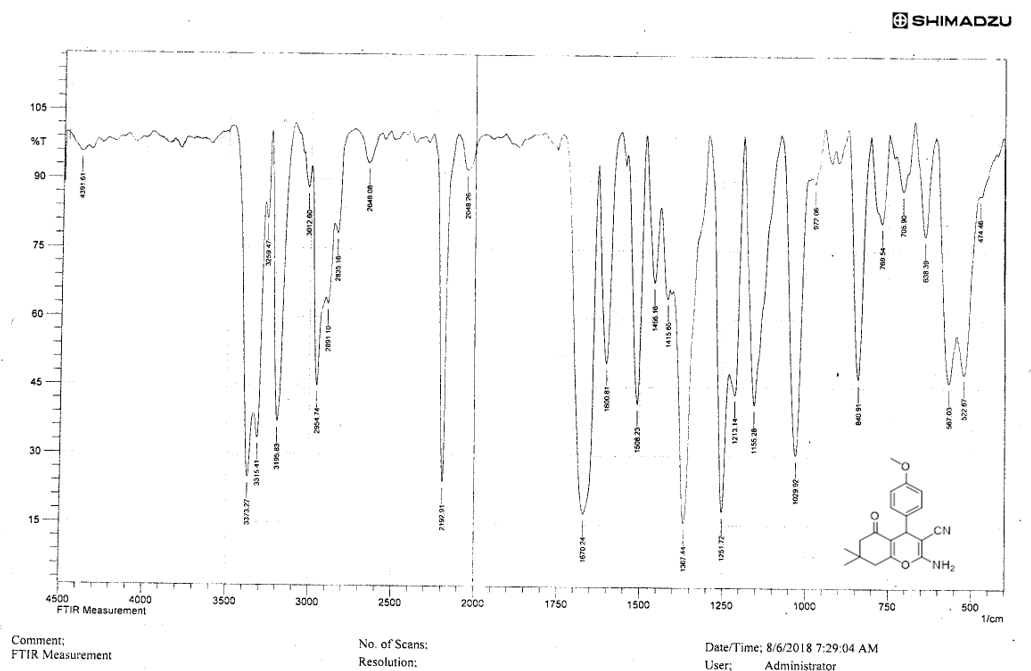


**Figure S11.** FT-IR spectrum of product (**4k**)


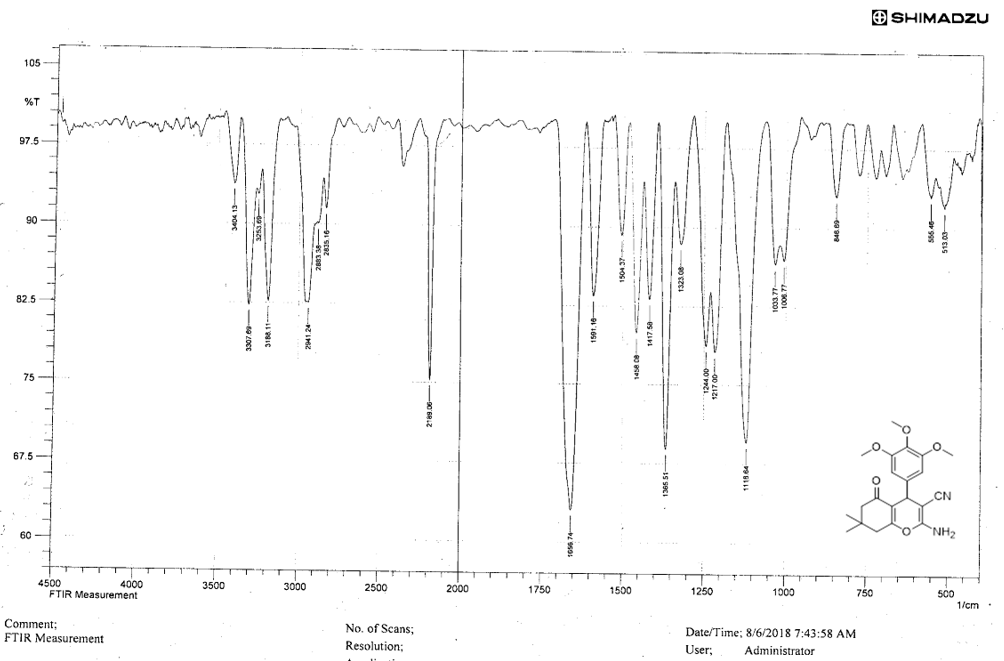


**Figure S12.** FT-IR spectrum of product (**4l**)


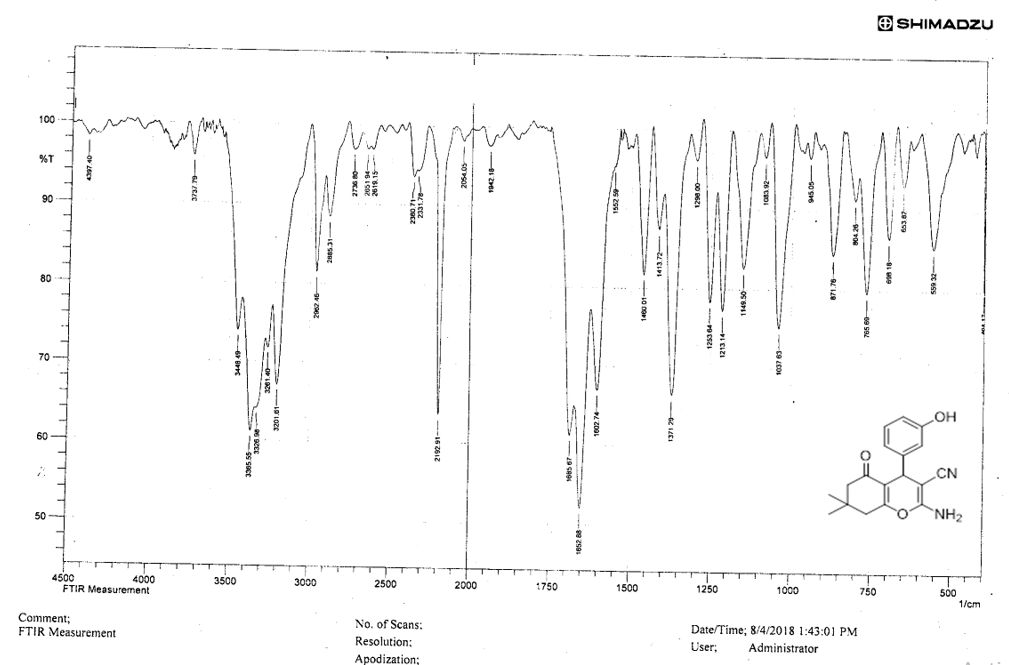


**Figure S13.** FT-IR spectrum of product (**4m**)


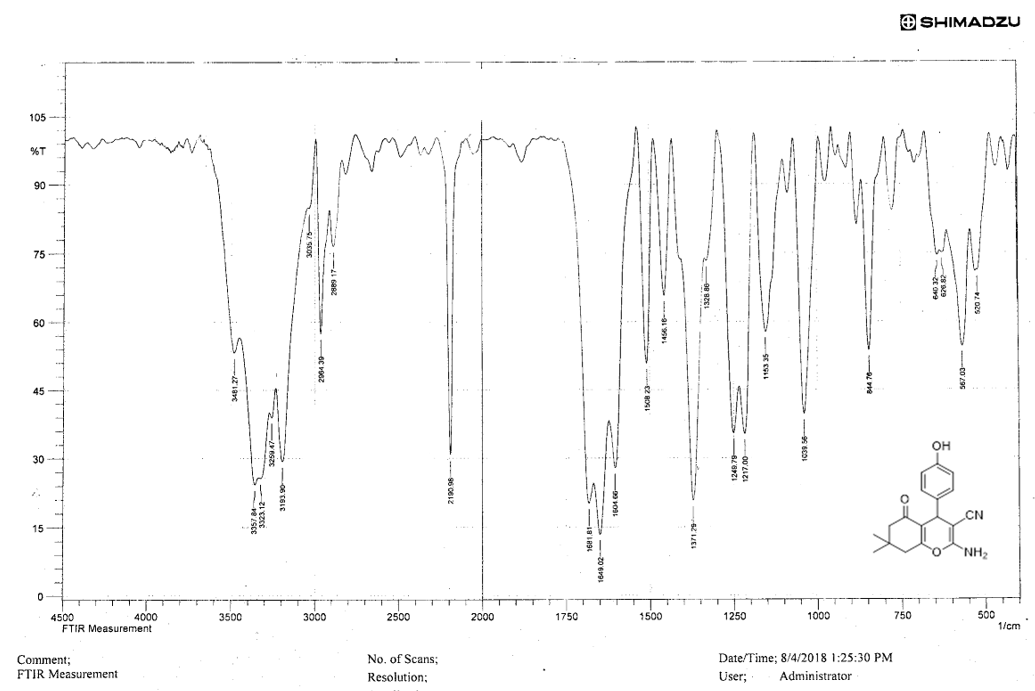


**Figure S14.** FT-IR spectrum of product (**4n**)


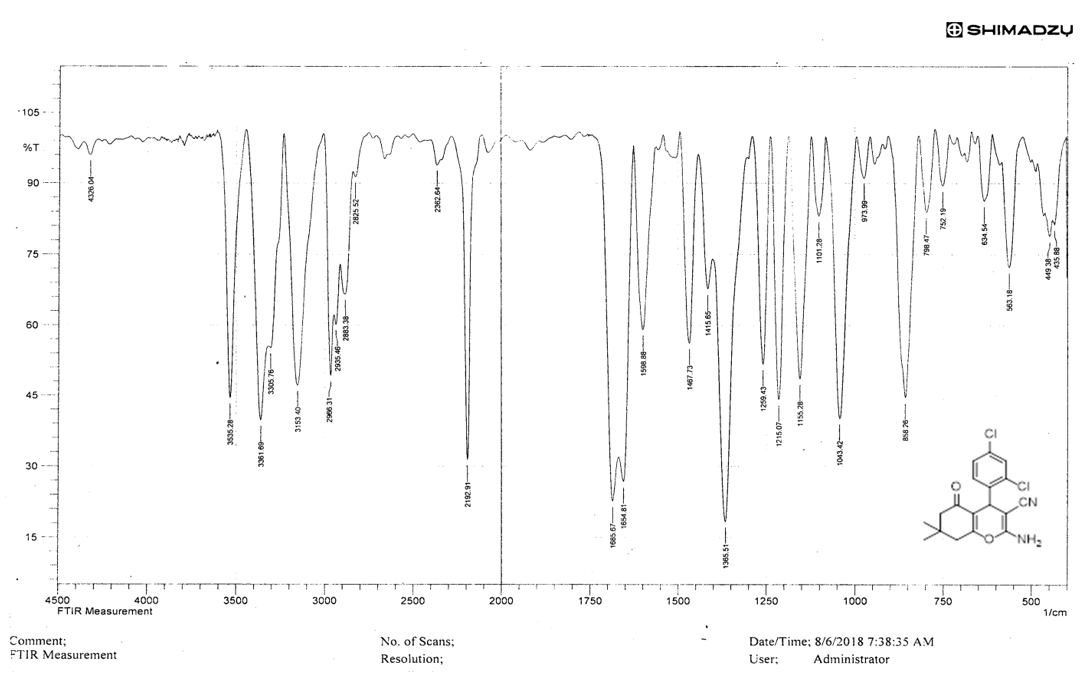


**Figure S15.** FT-IR spectrum of product (**4o**)


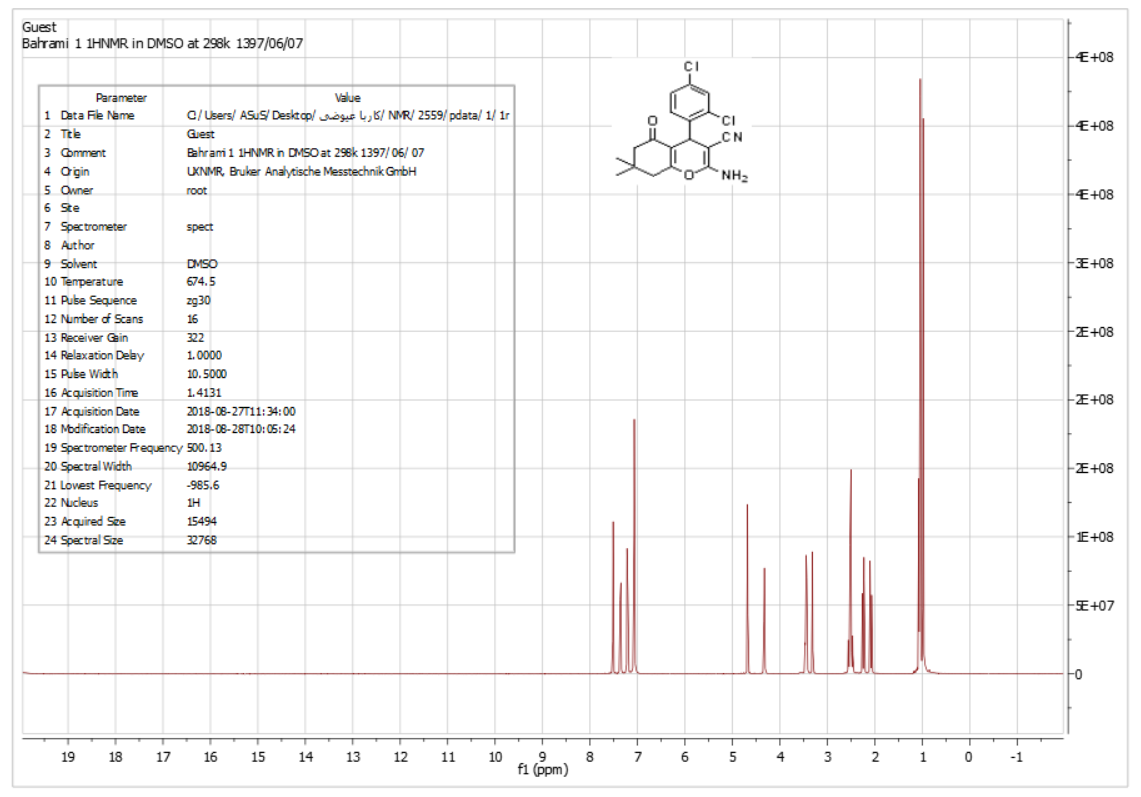


**Figure S16.** ^1^H NMR spectrum of product (**4o**)


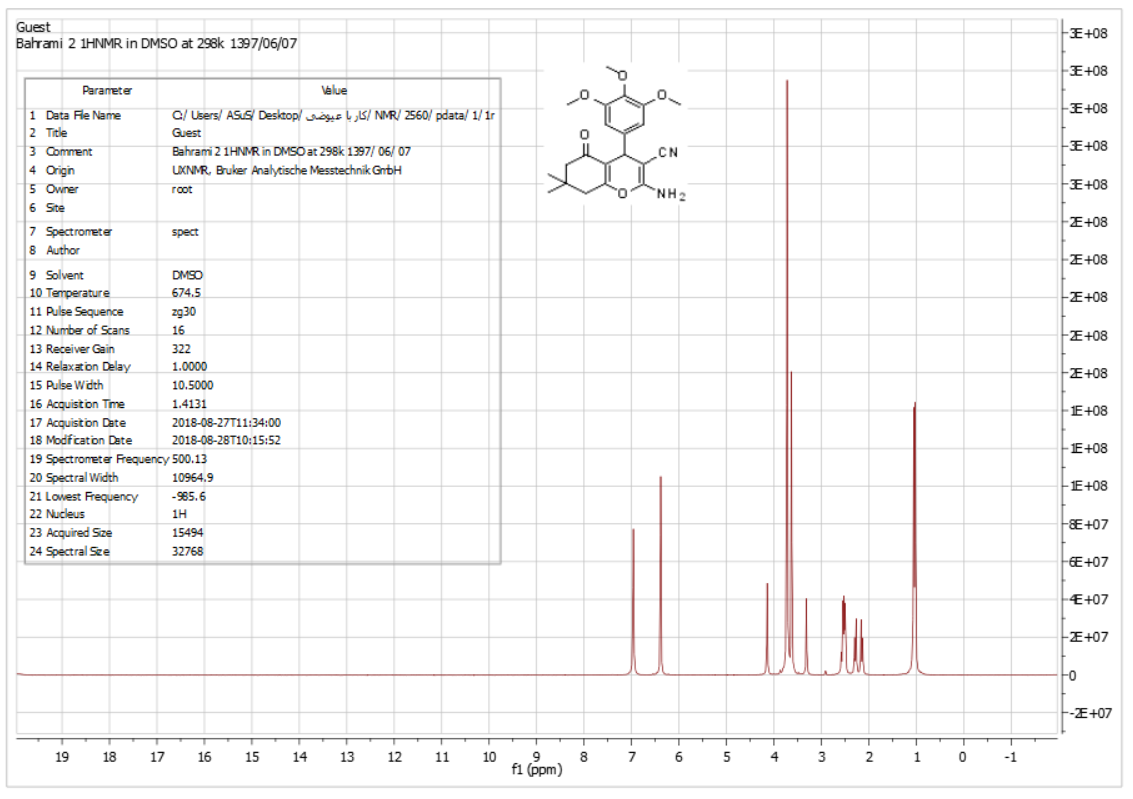


**Figure S17.** ^1^H NMR spectrum of product (**4l**)


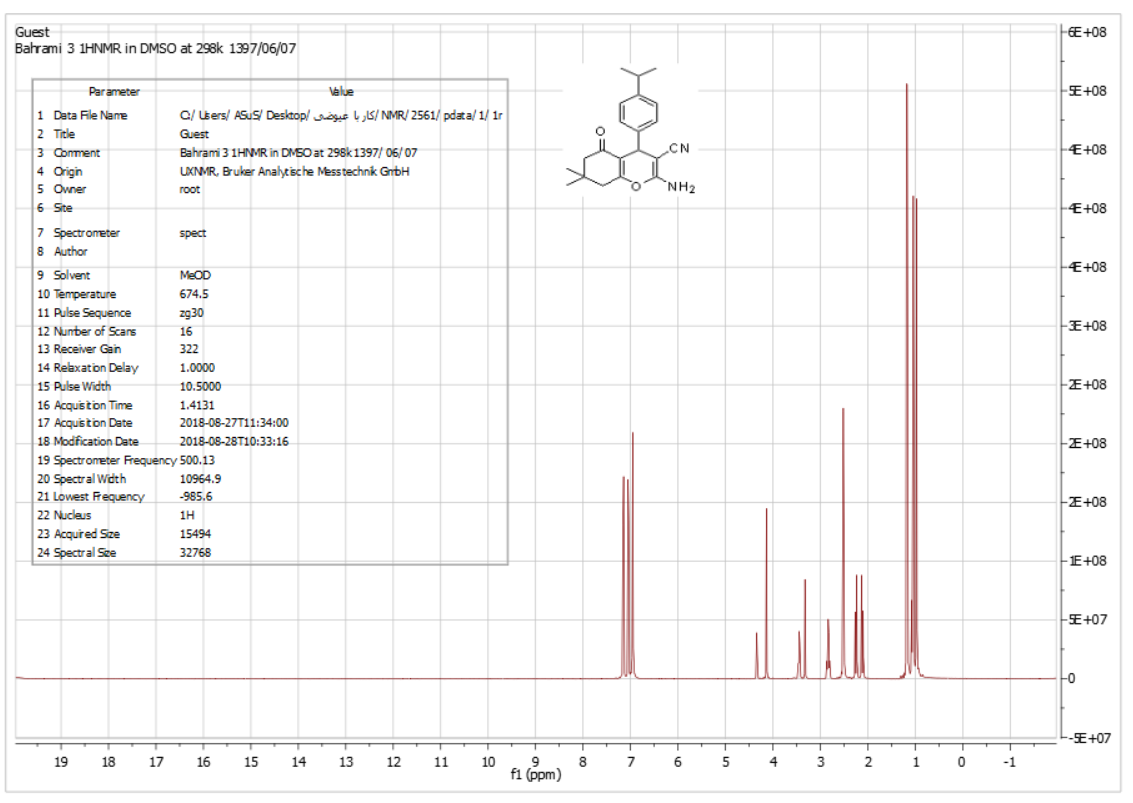


**Figure S18.** ^1^H NMR spectrum of product (**4h**)


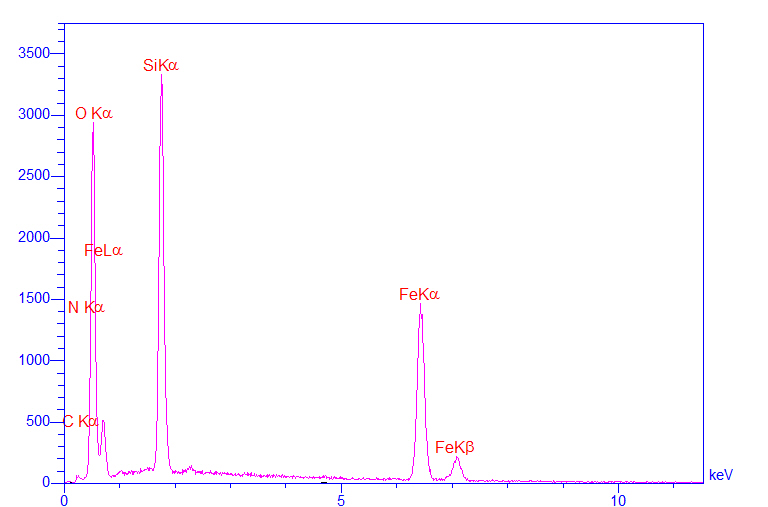


**Figure S19.** EDX analysis of the reused catalyst


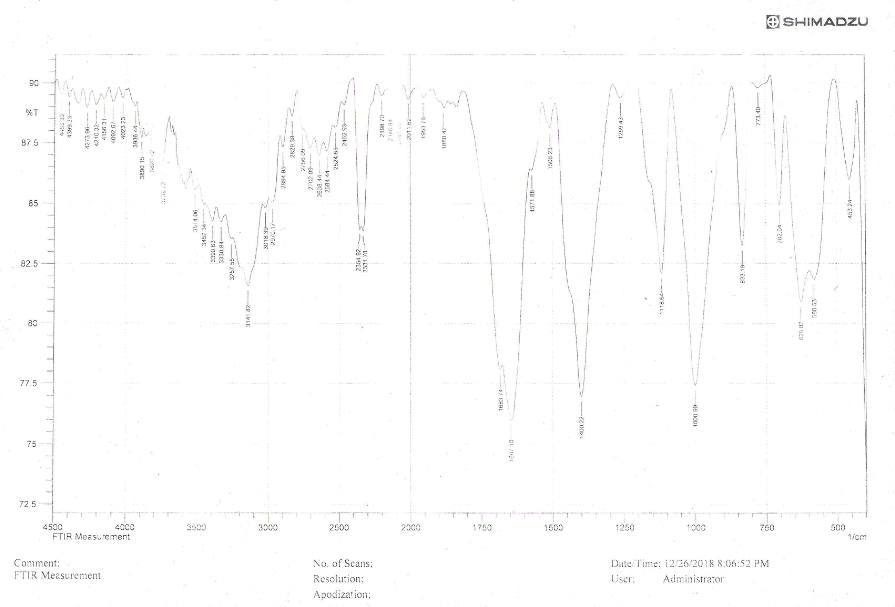


**Figure S20.** FT-IR spectrum of the reused catalyst.
